# A Facile Synthesis of Novel Amorphous TiO_2_ Nanorods Decorated rGO Hybrid Composites with Wide Band Microwave Absorption

**DOI:** 10.3390/nano10112141

**Published:** 2020-10-27

**Authors:** Hao Zhang, Yongpeng Zhao, Xuan Yang, Guolin Zhao, Dongmei Zhang, Hui Huang, Shuaitao Yang, Ningxuan Wen, Muhammad Javid, Zeng Fan, Lujun Pan

**Affiliations:** 1 School of Physics, Dalian University of Technology, Dalian 116024, China; zhangh0225@mail.dlut.edu.cn (H.Z.); zhaoyp13@mail.dlut.edu.cn (Y.Z.); zhangdm08@163.com (D.Z.); hhhalovelva@gmail.com (H.H.); 1203@mail.dlut.edu.cn (S.Y.); wenningxuan1119@mail.dlut.edu.cn (N.W.); Javidssp908@gmail.com (M.J.); fanzeng@dlut.edu.cn (Z.F.); 2 School of Microelectronics, Dalian University of Technology, Dalian 116024, China; 3 School of Materials Science and Engineering, Dalian University of Technology, Dalian 116024, China; yangxuan_1@mail.dlut.edu.cn; 4 School of Physics and Astronomy, Shanghai Jiao Tong University, Shanghai 200240, China; zhaogl17@sjtu.edu.cn

**Keywords:** amorphous TiO_2_, rGO, microwave absorption, complex permittivity, impedance matching, quarter-wavelength matching model

## Abstract

Amorphous structures may play important roles in achieving highly efficient microwave absorption performance due to the polarization losses induced by the disorders, vacancies and other functional groups existed in them. Herein, a kind of amorphous TiO_2_/rGO composite (a-TiO_2_/rGO) was successfully fabricated via a facile one-step solvothermal method. The complex permittivity of the composites can be regulated by adjusting the addition of precursor solution. The minimum reflection loss of a-TiO_2_/rGO composites reached −42.8 dB at 8.72 GHz with a thickness of 3.25 mm, and the widest efficient absorption bandwidth (EAB) was up to 6.2 GHz (11.8 to 18 GHz) with a thickness of only 2.15 mm, which achieved the full absorption in Ku band (12 to 18 GHz). Furthermore, the EAB was achieved ranging from 3.97 to 18 GHz by adjusting the thickness of the absorber, covering 87.7% of the whole radar frequency band. It is considered that the well-matched impedance, various polarization processes, capacitor-like structure and conductive networks all contributed to the excellent microwave absorption of a-TiO_2_/rGO. This study provides reference on constructing amorphous structures for future microwave absorber researches and the as-prepared a-TiO_2_/rGO composites also have great potential owing to its facile synthesis and highly efficient microwave absorption.

## 1. Introduction

Nowadays, with the rapid development of electronic information technology, electromagnetic radiation and pollution originating from electronic devices pose a threat to human health and information safety [1,2,3,4,5,6] and consequently the design and fabrication of microwave absorption materials which can dissipate microwave energy efficiently has become an urgent requirement [7,8,9]. In recent years, extensive efforts on the design of microwave absorbers have been focused on strengthening reflection loss (RL), broadening absorption bandwidth and reducing weight as well as thickness. Various microwave absorption materials based on carbon nanotubes (CNTs) [10,11,12], graphene [13,14,15], magnetic nanoparticles [16,17,18], conductive polymer [19,20], MXene [21,22,23], etc., have been developed, exhibiting promising microwave absorption performance.

Among these materials, graphene is expected to be one of the most promising candidates owing to its advantages of light weight, stable physical and chemical properties, low density, high specific surface area and remarkable electron mobility [24,25,26]. Unfortunately, the excessively high electrical conductivity of graphene always leads to a high reflection toward the incident microwave, which further results in the poor impedance matching [14,27,28]. On the other hand, the limited loss mechanism also leads to the weak microwave absorption of graphene [29,30]. To solve these drawbacks, many studies have combined graphene with other nanomaterials such as metals/alloys [31,32], ferrites [6,33,34], metal oxides/sulfide [35,36,37] and so on, which could achieve high magnetic or dielectric losses and thus add more loss mechanisms to improve their microwave absorption performance. These results indicate that their microwave absorption properties were indeed improved after introducing these materials. However, the addition of loss mechanism inevitably increases the complexity and difficulties of synthesis process, which limits the further practical applications of these materials. Meanwhile some shortcomings such as the poor physical or chemical stability, aggregation and high density of the introduced particles also become new challenges for the design of microwave absorption materials. Furthermore, compared with graphene, reduced graphene oxide (rGO) possesses more defects and functional groups which would achieve the synergistic effects of polarization loss and conduction loss. The rGO also exhibits relatively low electrical conductivity which would facilitate the impedance matching of the absorber. Therefore, it is feasible to choose rGO to add more loss mechanisms for achieving efficient microwave absorption.

Recently, dielectric materials have attracted considerable attention in microwave absorption owing to their thermal and chemical stabilities and high dielectric losses. For example, Chen et al. fabricated polyoxometalate-based materials [38] and Cu_9_S_5_ nanonets [39], both of which achieved excellent microwave absorption performance. As a typical dielectric material, titanium dioxide (TiO_2_) has been reasonably considered to be a promising candidate for microwave absorption materials. Mo et al. [40] have fabricated a porous CNT@TiO_2_ sponge, achieving a minimum RL value of −31.8 dB with the widest efficient absorption bandwidth (EAB) of 2.76 GHz. However, the microwave absorption performance of pure TiO_2_ seems not as good as expected because of the single dielectric loss mechanism. Thus, many studies were performed to add extra materials to TiO_2_ or to treat TiO_2_ with an additional hydrogenation process to achieve more loss mechanisms, which complicated the fabrication of the absorber and made it harder to obtain a lightweight material. Recently, constructing an amorphous structure with multiple defects have been proved to be an effective method to induce various polarizations by microwaves [41]. For example, Shen et al. [42] have developed a kind of amorphous FeCo nanowires, which showed a minimum reflection value of −25.88 dB with an EAB of 5.40 GHz. It is thought that amorphous structures would be an effective way to enhance the microwave absorption performance of TiO_2_. Moreover, amorphous TiO_2_ (a-TiO_2_) exhibits poor electric conductivity compared with graphene, further inspiring the strategy that a-TiO_2_ can be combined with graphene to adjust the excessively high electric conductivity of graphene. Therefore, the impedance matching property of the composite could also be controlled easily via changing the proportion of two components. In addition, considering the morphology of the nanomaterials, 1D nanostructures including nanowires, nanotubes and nanorods possess higher aspect ratio as well as shape anisotropy, which increases their interfacial polarization area and improves charge transportation along the axial direction, further enhancing the microwave absorption performance. Consequently, designing a 1D nanostructure with a-TiO_2_ would be a promising strategy for microwave absorption.

In this work, a-TiO_2_ nanorods were uniformly implanted in reduced graphene oxidize (rGO) nanosheets via a facile one-pot solvothermal method. The impedance matching properties of the composites could be easily adjusted by changing the proportion of a-TiO_2_ components. It was found that the as-prepared a-TiO_2_/rGO composites exhibited excellent microwave absorption performance with light weight, thin thickness, strong microwave absorption and wide EAB. Significantly, the microwave absorption performance became weaker after converting the a-TiO_2_ into crystalline TiO_2_ (c-TiO_2_) in the composites, indicating the superiority of amorphous structure in the high-performance microwave absorbers.

## 2. Materials and Methods

### 2.1. Materials

Graphene oxide (GO) was purchased from XFNANO (Nanjing, China). Tetrabutyltitanate (TBT), glycerol (C_3_H_8_O_3_), and ethyl alcohol absolute (C_2_H_5_OH, wt% ≥ 99.7%) were purchased from Tianjin Kermel Chemical Reagent Co., Ltd. (Tianjin, China). All reagents were analytical grade and not further purified. All water used in experiments was deionized water.

### 2.2. Synthesis of a-TiO_2_/rGO Composites

**Figure 1:**
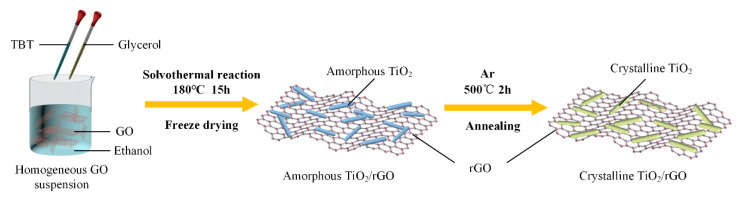
Schematic illustration of formation process for a-TiO_2_/rGO and c-TiO_2_/rGO composites.

The schematic illustration of formation process for a-TiO_2_/rGO and c-TiO_2_/rGO composites is shown in Figure 1. The a-TiO_2_/rGO composites were prepared via a facile one-pot solvothermal method. Initially, 75 mg GO was dispersed in 25 mL ethyl alcohol, followed by a 2 h sonication treatment to form a homogeneous dispersion. Then 5 mL glycerol and 0.25 mL TBT were added into the dispersion in sequence. After another sonication treatment for 30 min, the mixture was transferred into a 50 mL Teflon-lined autoclave and heated at 180 °C for 15 h. The precipitate in the resulting product was collected with suction filtration, washed with ethanol for 4–5 times and then was freezedried for 24 h. Finally, the a-TiO_2_/rGO composite was obtained. Repeatedly, the similar composites were prepared by using different TBT contents of 0.25, 0.5, and 1 mL, denoted by a-T1, a-T2, and a-T3, respectively.

### 2.3. Synthesis of c-TiO_2_/rGO Composites

As a comparison, the as prepared a-TiO_2_/rGO composites were converted into c-TiO_2_/rGO composites by an annealing method. The a-TiO_2_/rGO powders were heated to 500 °C with a ramping rate of 5 °C/min and maintained at 500 °C for 2 h under a high purity argon gas (Ar, 350 sccm, purity ≥ 99.999%) atmosphere. After the annealing treatment, the samples of a-T1, a-T2, and a-T3 were relabeled as c-T1, c-T2, and c-T3, respectively.

### 2.4. Characterizations

The morphologies of as-prepared samples were observed by a field emission scanning electron microscopy (SEM, NanoSEM450, FEI, Hillsboro, OR, USA) equipped with an energy dispersive X-ray spectroscopy (EDS). Besides, transmission electron microscopy (TEM, Tecnai F30, FEI, Hillsboro, OR, USA) was also used to examine the microstructural features. X-ray diffraction (XRD, Lab XRD-7000s, Shimadzu, Kyoto, Japan) was performed to characterize the amorphous and crystalline components in these composites with a Cu Kα radiation source. The banding energy was measured by X-ray photoelectron spectroscopy (XPS, ESCALAB250Xi, Thermo Fisher Scientific, Waltham, MA, USA) with an Al Kα radiation. For electromagnetic characterizations, the as-prepared composites (20 wt%) were uniformly mixed with melted paraffin (80 wt%) and then pressed into a toroidal shape with dimensions of external diameter of 7.00 mm, inner diameter of 3.04 mm and thickness of 2.0 mm. Then the complex permittivity and relative complex permeability of the composites were measured via a network analyzer (8720B, Agilent, Santa Clara, CA, USA) in the band of 2 to 18 GHz. For the sample of a-T2, we prepared another two paraffin composites containing a-T2 of 10 wt% and 15 wt% to investigate the best mass ratio for microwave absorption.

## 3. Results and Discussion

### 3.1. Morphological and Structural Analysis

**Figure 2:**
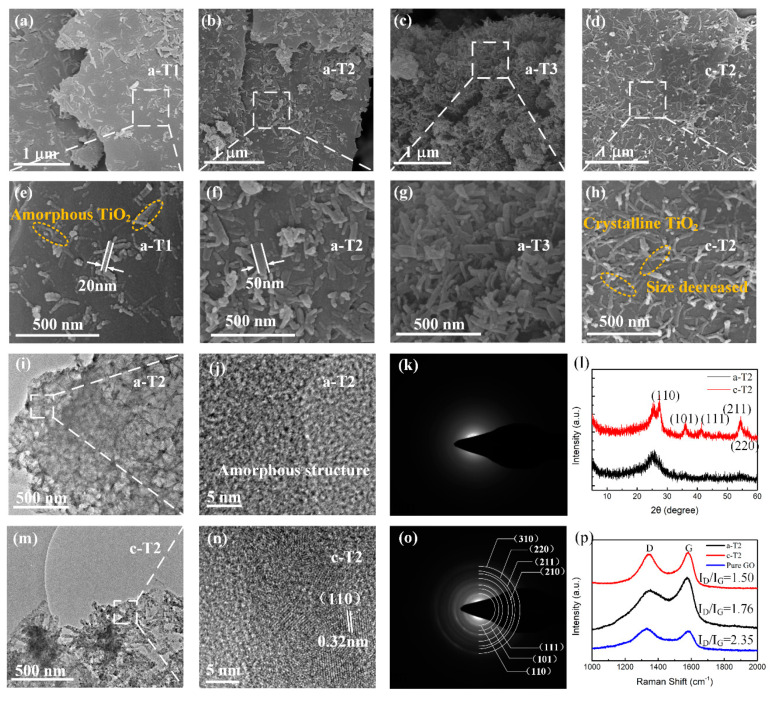
SEM images of (**a**,**e**) a-T1, (**b**,**f**) a-T2, (**c**,**g**) a-T3 and (**d**,**h**) c-T2; (**i**,**m**) TEM images, (**j**,**n**) HRTEM micrographs, (**k**,**o**) SAED patterns, (**l**) XRD patterns of a-T2 and c-T2; (**p**) Raman spectra of a-T2, c-T2 and pure GO.

Figure 2 shows the SEM images of a-TiO_2_/rGO and c-TiO_2_/rGO. The as-prepared TiO_2_/rGO composites consist of TiO_2_ nanorods with diameters of 20–50 nm and lengths up to 100–300 nm, which are uniformly implanted on rGO nanosheets. In Figure 2a, the density of TiO_2_ nanorods is relatively low because of the low amount of TBT added. It should be noted that most of the TiO_2_ nanorods attach parallel to the rGO nanosheets to add more interface polarization sites, which is conductive to microwave absorption. From Figure 2a–c, the increase in density of TiO_2_ nanorods on rGO nanosheets can be clearly observed with the increase of addition amount of TBT, suggesting that the density of TiO_2_ nanorods can be well controlled by adjusting the addition amount of TBT. As shown in Figure 2d,h, the rGO nanosheets undergo no apparent changes while the diameter of TiO_2_ nanorods decreases after the annealing treatment, which might be explained as the volume contraction of TiO_2_ nanorods caused by the decrease of disorders and vacancies [43]. TEM images in Figure 2 show the microstructures of a-TiO_2_/rGO and c-TiO_2_/rGO. It is observed from Figure 2i,m that the TiO_2_ nanorods are uniformly dispersed on the rGO nanosheets, which further confirms the results of the SEM observations. The HRTEM images shown in Figure 2j reveal no distinct lattice fringe, confirming the amorphous structure of a-TiO_2_/rGO, while Figure 2n presents the crystal structure of TiO_2_. The interplanar spacing is 0.32 nm, which corresponds to the (110) crystal plane of TiO_2_. Moreover, the selected-area electron diffraction (SEAD) patterns in Figure 2k,o show a blurred image for a-TiO_2_/rGO and clear diffraction rings for c-TiO_2_/rGO, respectively, which is consistent with the HRTEM results.

Figure 2l shows the XRD spectra of a-TiO_2_/ rGO and c-TiO_2_/ rGO. A main broad diffraction peak at around 26.0° is observed in all samples, which corresponds to the (002) crystal plane of graphite carbon. However, no other obvious characteristic diffraction peak from the a-T2 sample, indicating an amorphous TiO_2_ structure. After an annealing treatment, a series of clearer diffraction peaks appears at 27.4°, 36.1°, 41.3°, 54.3° and 56.7°, which were well matched with (110), (101), (111), (211) and (220) crystal planes of rutile phase TiO_2_ (PDF file #01-086-0147, P42/mnm, a = b = 4.594 Å and c = 2.958 Å), respectively. As shown in the XRD patterns, both a-T2 and c-T2 shows no obvious peak around 10°, indicating that most of the GO is reduced. Moreover, the diffraction rings in Figure 2o also agree well with the XRD pattern of c-T2. These results reveal that amorphous TiO_2_ has been transformed into crystalline TiO_2_ via the annealing treatment.

Changes in the graphitization degree of GO by annealing treatment are also demonstrated by Raman spectroscopy. As shown in Figure 2p, both a-T2 and c-T2 present two prominent peaks around 1575 cm^−1^(G band) and 1340 cm^−1^(D band), which denote the vibrations of sp2 hybridization, and the vibration of disordered graphitic lattice [44]. Herein, the G band and D band signals mainly originated from the graphite carbon atoms as well as the disordered structures, and a small part of signals were derived from the organic materials synthesizing TiO_2_ such as TBT and glycerol. Thus, the intensity ratio (I_D_/I_G_) was calculated to demonstrate the disorder degree of the carbon materials. It is observed that the pure GO possesses the highest I_D_/I_G_ value of 2.35, indicating that a large number of defects and oxygen-containing functional groups exist in pure GO [45]. In addition, the I_D_/I_G_ value of a-T2 is 1.76 while the I_D_/I_G_ value of c-T2 is 1.50. Therefore, we believe that the rGO existing in a-T2 is partially reduced. That is to say, the partially reduced rGO not only provides enough conduction loss due to the improvement of graphitization degree, but also retains some defects and oxygen-containing functional groups to add more polarization loss, which is exactly what we expected to achieve.

**Figure 3:**
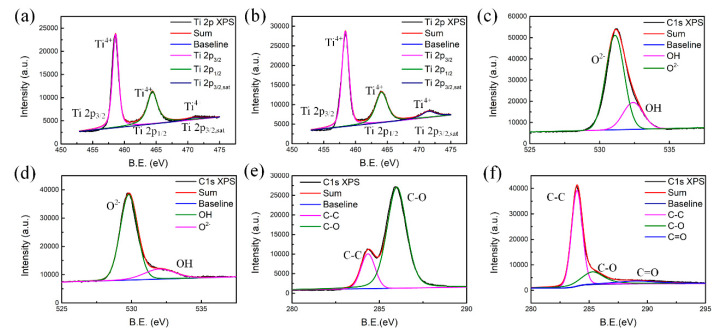
XPS spectra of (**a**,**b**) Ti 2p, (**c**,**d**) O 1s, and (**e**,**f**) C 1s of a-T2 and c-T2.

The X-ray photoelectron spectroscopy (XPS) spectra of a-T2 and c-T2 are shown in Figure 3, where the Ti 2p spectra are similar for a-T2 and c-T2, as they both showed almost identical pattern from lattice Ti^4+^ ions with peaks centering at 458.6, 464.4, and 471.6 eV (Figure 3a,b). The O 1s spectra (Figure 3c,d) reveal that the c-T2 has less content of chemisorbed oxygen and some oxygen-containing functional groups such as OH, because its intensity around 532.5 eV is smaller than that of a-T2. It should be noticed that the O 1s peaks at 530–531 eV could be attributed to the lattice oxygen for TiO_2_ [46]. After annealing treatment, this peak tended to shift by approximately 1 eV to the lower energy side due to crystallization. In addition, it is observed from the C 1s XPS spectra (Figure 3e,f) that a sharp decrease in the relative intensity of the C-O peak around 285.9 eV after annealing, indicating the reduction of oxidized carbon atoms. The lack of oxygen-containing functional groups and the reduction of oxidized carbon atoms in c-T2 can be confirmed further with Fourier transform infrared spectroscopy (FTIR) results (Figure S1). Compared with the clear peaks in a-T2, the vibration intensities of -OH (~3390 cm^−1^, ~1038 cm^−1^), C-O (~1117 cm^−1^, ~810 cm^−1^), and C=O (~1730 cm^−1^, ~605 cm^−1^) bonds in c-T2 all become smaller. Thus, it is reasonable to conclude that both oxygen-containing functional groups and defects are decreased by the annealing treatment, and the polarization losses induced by these groups and defects are decreased sequentially [47,48,49].

### 3.2. Microwave Absorption Properties

**Figure 4:**
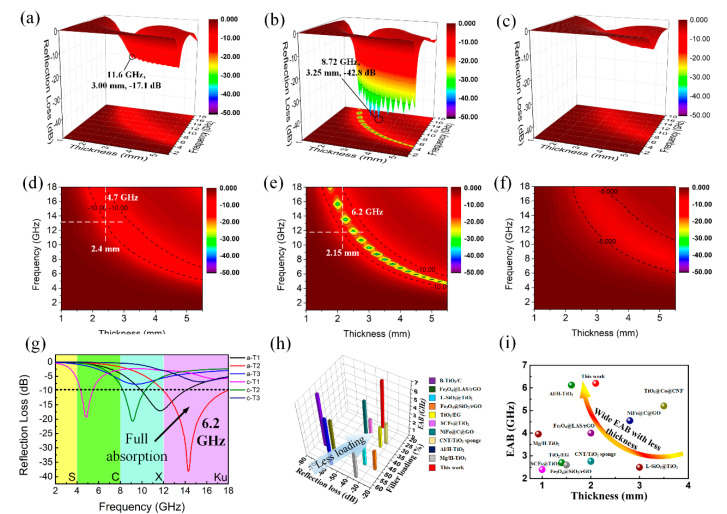
3D RL and 3D projection plots of (**a**,**d**) a-T1, (**b**,**e**) a-T2 and (**c**,**f**) a-T3; (**g**) the maximum EABs of the TiO_2_/rGO composites; comparison of (**h**) the filler loading and (**i**) the matching thickness of the maximum EAB with graphene- and TiO_2_-based absorbers reported in other researches.

Generally, the RL represents the microwave absorbing efficiency of materials. A RL value less than −10 dB indicates that more than 90% of the incident wave have been absorbed and the corresponding absorption frequency range with RL less than −10 dB is considered as effective bandwidth. According to the transmission line theory [50,51,52], the RL value of an absorber is expressed as follows:

(1) $$Z_{in}=Z_{0}\sqrt{\frac{\mu_{r}}{\varepsilon_{r}}}\tan h\left(j\frac{2\pi fd\sqrt{\mu_{r}\varepsilon_{r}}}{c}\right)$$

(2) $$\mathrm{RL}\left(\mathrm{dB}\right)=20\log\left|\frac{Z_{in}-Z_{0}}{Z_{in}+Z_{0}}\right|$$

where *ε*_r_ and *μ_r_* are the complex permittivity and permeability, respectively; *f* is the frequency of incident microwave; *d* is the thickness of absorber; *c* is the velocity of light; *Z*_0_ refers to the input impedance of the free space; and *Z_in_* is the input impedance of absorber. Firstly, in view of the possible influence of different filler loading ratios on microwave absorption performance, we measured the electromagnetic parameters and calculated the frequency-dependent RL values of a-T2 with filler loading ratios of 10 wt%, 15 wt% and 20 wt%. From Figure S2, it is observed that a-T2 with the loading ratio of 20 wt% has the minimum reflection loss (RL) value, indicating its best microwave absorption performance. Therefore, filler loading of 20 wt% is selected as the optimal value in the following measurements. Figure 4 and Figure S3 illustrate the 3D RL with projection plots of a-T1, a-T2, a-T3, c-T1 and c-T2. The minimum RL value for a-T1 (Figure 4a,d) reaches −17.1 dB at 11.6 GHz when the thickness is 3 mm and the widest EAB is 4.7 GHz, which is much better than pure rGO. For a-T2 (Figure 4b,e), the minimum RL value reaches −42.8 dB at 8.72 GHz when the thickness is 3.25 mm and the widest EAB is 6.2 GHz, showing the best microwave absorption properties. However, it is observed from Figure 4c,f that the RL of a-T3 does not reach −10 dB in all frequency range of 2 to 18 GHz. These results suggest that the microwave absorption properties of a-TiO_2_/rGO composites can be easily adjusted by changing the amorphous TiO_2_ ratios. In Figure S3a,b, the widest EAB of c-T1 declines obviously compared with a-T1, indicating that the c-TiO_2_ has weaker capability to adjust the impedance matching effectively. The little-changed RL values are possibly because that the TiO_2_ ratio in c-T1 is so small that the polarization loss induced by the defects of a-TiO_2_ is negligible. Figure S3c,d demonstrate that both the minimum RL value and the widest EAB of c-T2 decline sharply compared with a-T2, because the decrease of polarization sites and the impedance mismatching lead to the degradation of microwave absorption performance. As shown in Figure 4g, a-T2 obtains the widest EAB of 6.2GHz among all the TiO_2_/rGO composites, achieving the full absorption of Ku band, which should be credited to its excellent impedance matching. What is more, Figure 4h,i and Table S1 show that the a-T2 not only possesses the minimum RL value and wide EAB, but also exhibits the advantages of less filler loading and less matching thickness compared with the graphene- and TiO_2_-based absorbers reported in other researches [40,53,54,55,56,57,58,59,60,61,62].

### 3.3. Analysis of Electromagnetic Parameters

**Figure 5:**
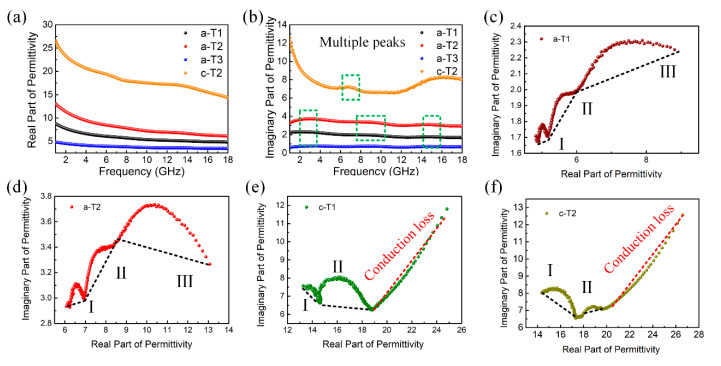
(**a**) Real permittivity, (**b**) imaginary permittivity of a-T1, a-T2, a-T3 and c-T2 at 2-18 GHz; Cole-Cole plots of (**c**) a-T1, (**d**) a-T2, (**e**) c-T1 and (**f**) c-T2.

It is widely accepted that the microwave absorption properties of an absorber are strongly determined by its electromagnetic parameters including complex permittivity (*ε_r_* = *ε*′ − *jε*″) and complex permeability (*μ_r_* = *μ*′ − *jμ*″). The measured electromagnetic parameters for a-T1, a-T2, a-T3 and c-T2 are shown in Figure 5 and Figure S4. The real parts of complex permittivity (*ε*′) and complex permeability (*μ*′) represent the storage capability of electric and magnetic energies, while the imaginary parts (*ε*″ and *μ*″) stand for the loss capability of electric and magnetic energies [63]. Figure S4a,b show that the *μ*′ and *μ*″ values range from 0.96 to 1.10 and 0 to 0.06, respectively, indicating their weak magnetic properties. This means that the complex permittivity dominates the microwave absorption. From Figure 5a, it is observed that the real part of complex permittivity for all the samples decrease with the increase of microwave frequency. However, the imaginary parts shown in Figure 5b have multiple peaks, which are ascribed to polarizations. Both *ε*′ and *ε*″ values first increase then decrease with the increase of TiO_2_ component, indicating that moderate TiO_2_ nanorods prevent the stacking of rGO nanosheets and thus improve the electron transfer capability of the composite [64,65,66]. Furthermore, a-T2 processes larger *ε*′ and *ε*″ values than a-T1 and a-T3, revealing that the a-T2 absorbs more electric energy from the electromagnetic field, possibly owing to its more polarizable groups [67]. These mean that a-T2 is more efficient in dissipating the electric field energy. However, excessive TiO_2_ would nearly wrap the rGO nanosheets and thus decline the electron transfer capability of the composite, resulting in the smallest *ε*′ and *ε*″ value of a-T3.

However, it is observed from Figure 5a,b that both *ε*′ and *ε*″ values of c-T2 increase compared with a-T2. The electromagnetic parameters for c-T1 and c-T3 shown in Figure S5 also demonstrate the similar changes compared with c-T2. These changes are mainly caused by that the annealing treatment not only improves the crystalline degrees of TiO_2_ and rGO, but also removes the oxygen-containing functional groups and defects which are barriers or traps for the transport of electrons. On one hand, the improvement of crystalline degrees makes it hard to adjust the impedance matching of the composites. On the other hand, the removement of functional groups and defects also decrease the polarization process, which leads to the recession of microwave absorption performance.

Furthermore, these results are confirmed by Cole-Cole semicircles according to the Debye theory [68], from which the *ε*′ and *ε*″ can be expressed as:

(3) $$\varepsilon^{\prime }=\varepsilon_{\infty }+\left(\varepsilon_{s}-\varepsilon_{\infty }\right)/\left(1+\omega^{2}\tau^{2}\right),$$

(4) $$\varepsilon^{\prime \prime }=\omega \tau \left(\varepsilon_{s}-\varepsilon_{\infty }\right)/\left(1+\omega^{2}\tau^{2}\right),$$

(5) ω=2πf

where *f* represents the frequency of the microwave, ω represents the circular frequency of the microwave, *τ* is the polarization relaxation time, *ε*_s_ stands for the static permittivity, and $\varepsilon_{\infty }$ is the optical dielectric permittivity. According to these two equations, the relationship between ε′ and ε″ is deduced as:

(6) $${\left(\varepsilon^{\prime }-\frac{\varepsilon_{s}+\varepsilon_{\infty }}{2}\right)}^{2}+{\left(\varepsilon^{\prime \prime }\right)}^{2}={\left(\frac{\varepsilon_{s}+\varepsilon_{\infty }}{2}\right)}^{2}$$

Thus, it can be concluded that the plot of $\varepsilon^{\prime }$ versus $\varepsilon^{\prime \prime }$ is a single semicircle called Cole-Cole semicircle, each of which represents a Debye relaxation process. Figure 5c–f shows the $\varepsilon^{\prime }$-$\varepsilon^{\prime \prime }$ curves for a-T1, a-T2, c-T1 and c-T2. In Figure 5c,d, both a-T1 and a-T2 have three Cole-Cole semicircles corresponding to three Debye relaxation processes [69]. In a composite microwave absorber, interfacial polarization is generally considered to be the dominant polarization mechanism. With the additive amounts of a-TiO_2_, more free charges accumulate at the interfaces between rGO and TiO_2_, resulting in the Debye relaxation to transform electromagnetic energy to thermal energy. In addition, as shown in Figure 5e,f, not only does the amount of the Cole-Cole semicircles decrease, but also the size of the Cole-Cole semicircles becomes much smaller after the annealing treatment, which demonstrates less and weaker Debye relaxation processes because of the reduction of the defects and oxygen-containing functional groups. In addition, the straight-line part is related to the conduction loss, further confirming that annealing treatment improves the crystalline degrees of TiO_2_ and rGO.

To further investigate the microwave absorption properties of the composites, the dielectric and magnetic dissipation factors of $\mathrm{tg}\delta_{\varepsilon }=\varepsilon^{\prime \prime }/\varepsilon^{\prime }$ and $\mathrm{tg}\delta_{\mu }=\mu^{\prime \prime }/\mu^{\prime }$ are calculated, which provides a measure of how much power is dissipated in a material versus how much power is stored. And the value of attenuation constant α which determines the attenuation properties is also calculated via the following Equation [70]:

(7) $$\alpha =\frac{\sqrt{2}\pi f}{c}\sqrt{\left(\mu^{\prime \prime }\varepsilon^{\prime \prime }-\mu^{\prime }\varepsilon^{\prime }\right)+\sqrt{{\left(\mu^{\prime \prime }\varepsilon^{\prime \prime }-\mu^{\prime }\varepsilon^{\prime }\right)}^{2}+{\left(\mu^{\prime \prime }\varepsilon^{\prime }+\mu^{\prime }\varepsilon^{\prime \prime }\right)}^{2}}}$$

In addition, another key factor which affects the microwave absorption performance is the impedance matching of the materials. Impedance matching ($Z=Z_{in}/Z_{0}$) represents the ability of the incident wave to enter into the internal parts of absorbers. Theoretically, when the Z value is 1, all the incident waves are penetrating into the absorber without reflection, which is hard to realize for the whole frequency range of the microwave. Consequently, it is beneficial for improving microwave absorption performance to adjust the Z value close to 1.

**Figure 6:**
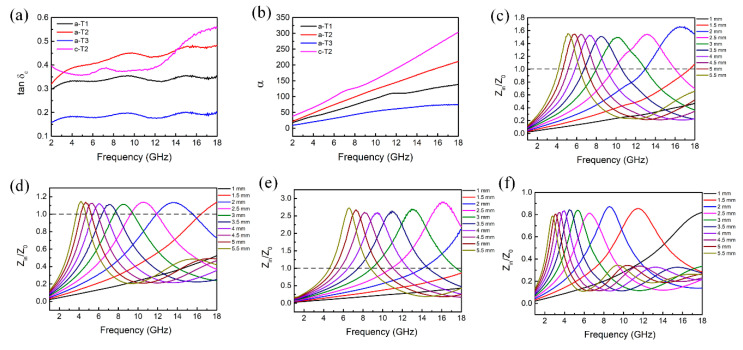
(**a**) Dielectric dissipation factor ($\mathrm{tg}\delta_{\varepsilon }$), (**b**) attenuation constant (α) of a-T1, a-T2, a-T3 and c-T2. Impedance matching (Z) with different thickness of (**c**) a-T1, (**d**) a-T2, (**e**) a-T3 and (**f**) c-T2 in the frequency range of 2-18 GHz.

As shown in Figure S6, the magnetic dissipation factor $\mathrm{tg}\delta_{\mu }$ for all the samples is around 0 with slight fluctuation, demonstrating the low magnetic losses of the composites. Furthermore, in Figure 6a, b, the a-T1 possesses smaller dielectric dissipation factor $\mathrm{tg}\delta_{\varepsilon }$ and attenuation constant α because of the lower content of TiO_2_. With the increase of TiO_2_ component, both the $\mathrm{tg}\delta_{\varepsilon }$ and α values increase initially then decrease, confirming that reasonable TiO_2_ content is beneficial to dissipate electromagnetic energy. However, compared with a-T2, the c-T2 exhibits lower $\mathrm{tg}\delta_{\varepsilon }$ value at most region from 2 to 18 GHz because the decrease of the polarization process while processes the highest α value due to the enhanced conductive loss. However, this effect also brings a negative influence on the impedance matching property. Figure 6c to f depict the frequency-dependent Z values of a-T1, a-T2, a-T3 and c-T2. It is well known that pure rGO possesses poor impedance matching because of its excessive permittivity. In Figure 6c, the Z value of a-T1 is around 1.5 at each impedance matching peak frequency (*f_z_*). Compared with pure rGO [71], the impedance matching becomes much better after the addition of TiO_2_. Especially, the a-T2 exhibits an optimal Z value of around 1.1 at each *f_z_* (Figure 6d), which is much closer to 1. 

From Figure 6e, it is found that the Z value of a-T3 at each *f_z_* far deviates from 1 because of the excessive addition of TiO_2_, indicating a poor impedance matching and poor microwave absorption performance. Furthermore, in Figure 6f, the Z values of c-T2 at all microwave frequency bands of 2 to 18 GHz deviate from 1, confirming that c-TiO_2_ could not do better in adjusting the impedance matching property compared with the a-TiO_2_. Therefore, it is concluded that a-T2 balances the relation between attenuation properties and impedance matching property excellently, implying its better microwave absorption properties.

**Figure 7:**
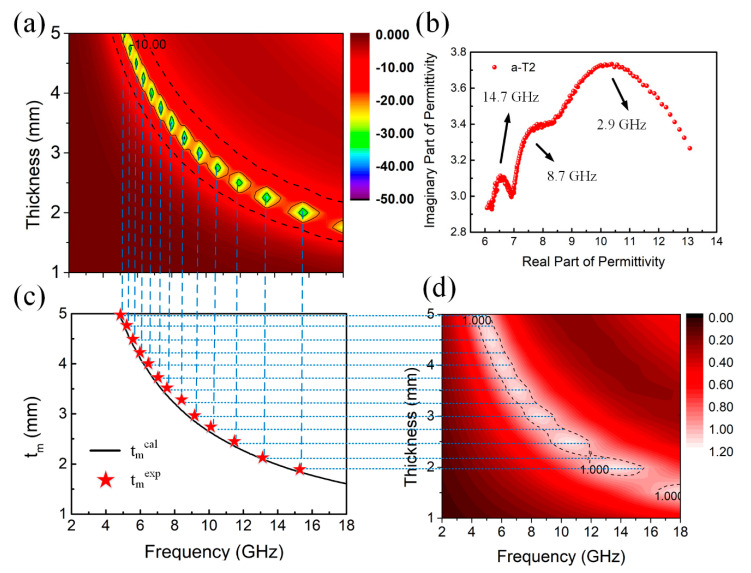
(**a**) 3D Frequency-dependent RL projection plots and (**b**) Cole-Cole plots of a-T2, (**c**) Simulations of the tm versus frequency for a-T2 under the quarter-wavelength matching model. (**d**) 3D Frequency-dependent Z projection plots of a-T2.

To better understand the microwave absorption performance of the a-T2, the quarter-wavelength matching model [72,73,74] is proposed to analyze the absorption mechanism of a-T2. In this model, the relationship between matching thickness (*t_m_*) and absorption peak frequency (*f_m_*) can be described by the following equation:

(8) $$t_{m}=\frac{nc}{4f_{m}}\frac{1}{\sqrt{\left|\varepsilon_{r}\right|\left|\mu_{r}\right|}};n=1,3,5,\ldots$$

where $\left|\varepsilon_{r}\right|$ and $\left|\mu_{r}\right|$ represent the modulus of complex permittivity and complex permeability respectively at matching frequency, and c is the velocity of light. Generally speaking, if the calculated *t_m_* value coincides with the experimental matching thickness, the phase cancellation effect will contribute to the microwave absorption. On one hand, it is clearly observed in Figure 7a that the absorption peaks of a-T2 shift to lower frequencies with the increase of thickness, which fits well to the quarter-wavelength matching model. 

On the other hand, it is also noticed from Figure 4 and Figure S3 that the absorption peaks of all the samples at the same matching thickness also shift to lower frequency regions after annealing treatment due to the increase of $\varepsilon_{r}$ values, which further confirms that the microwave absorption of the composites agrees well with the quarter-wavelength matching model. In addition, Figure 7c displays the relationship between *t_m_* and frequency for a-T2, in which the black line stands for the $t_{m}$ values calculated from the electromagnetic parameters (denoted as *t_m_^cal^*) and the pentagrams represent the experimental *t_m_* values (denoted as *t_m_^exp^*). Obviously, nearly all the *t_m_^exp^* values accord well with the calculated quarter-wavelength curve, further proving that the quarter-wavelength matching model dominates the relationship between *t_m_* and frequency for a-T2. Moreover, it is shown in Figure 7d that for each frequency where the impedance matching value equals 1, there is a corresponding thickness which also accords well with the calculated quarter-wavelength curve. Figure S7 shows the quarter wavelength matching model of several other samples, which indicates that the a-T2 model matches better among amorphous samples. In Figure S7c,d, although the practical matching thicknesses of crystalline samples are consistent with the simulated thicknesses, a lack of polarization and impedance mismatching restrict their microwave absorption. Figure 7b shows the Cole-Cole plot of a-T2, where three distinct semicircles are found around 2.9, 8.7 and 14.7 GHz, corresponding to the three peaks around 3, 8 and 14 GHz in *ε*″ curve of a-T2 in Figure 5b, indicating the existence of Debye relaxation process. In a word, the highly consistent thickness-frequency relationship among the RL values, quarter-wavelength curve and impedance matching properties determine the excellent performance of microwave absorption in a-T2.

### 3.4. Microwave Absorption Mechanisms

**Figure 8:**
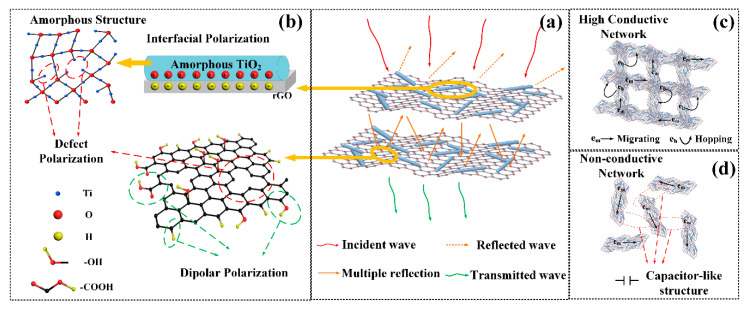
Schematic illustration of electromagnetic wave absorption mechanism of a-T2. (**a**) multiscattering loss, (**b**) polarizations loss, (**c**) conductive loss and (**d**) capacitor-like loss.

The overall results show that the a-T2 has the best microwave absorption performance. Firstly, a highly efficient microwave absorber should guarantee that the microwave irradiated on its surface can infiltrate into its internal parts as much as possible. As previously discussed, a-T2 balances the relation between electrically conductive rGO and dielectric TiO_2_ to achieve the best impedance matching, which extremely reduces the reflection of the incident microwave. Besides, as shown in Figure 7, the peak frequency of RL, the frequency where Z value equals to 1 and the frequency calculated from quarter-wavelength matching model accord well with each other, indicating a highly efficient absorption of incident microwave. It is certified that the quarter-wavelength matching is a crucial mechanism to determine the position of absorption peaks. Then the excellent $\mathrm{tg}\delta_{\varepsilon }$ value around the same peak frequency position guarantees the further dissipation of the incident microwave effectively. Figure 8 illustrates the possible dielectric loss mechanisms present in a-T2, which can be summarized as follows:

- Multiscattering loss: When the microwave propagates in the internal part of the absorber, it would be repeatedly scattered by the rGO nanosheets. Particularly, the TiO_2_ nanorods implanted in the rGO nanosheets further increase the scattering area and thus improve the multiscattering loss.
- Polarizations loss: On one hand, the defects and oxygen-containing functional groups in a-T2 would induce defect and dipole polarizations to improve the relaxation losses [75]. On the other hand, the accumulation of charges at the boundary between rGO and TiO_2_ would induce the interfacial polarizations and thus promotes the microwave absorption [76,77].
- Conductive loss: The adjacent rGO nanosheets contacting with each other establish an interconnected conductive network for electron transport. These electrons could migrate not only from one side of a rGO nanosheet to another, but also from one rGO nanosheet to another, which leads to significant conduction loss.
- Capacitor-like loss: Significantly, the capacitor-like structures [78,79] at the interfaces between non-contact rGO nanosheets could generate the induced charges, further enhancing the microwave absorption performance [80,81].

## 4. Conclusions

Amorphous TiO_2_/rGO (a-TiO_2_/rGO) composites have been successfully prepared via a facile one-step solvothermal method whereby the electromagnetic parameters of the composites can be adjusted easily by changing the TiO_2_ contents. The a-T2 balances the relation between conductive rGO and dielectric TiO_2_ excellently and thus exhibits the best microwave absorption performance with the minimum reflection loss of –42.8 dB achieved at 8.72 GHz. More importantly, the widest EAB of a-T2 reaches 6.2 GHz at 2.15 mm with the filler loading ratio of 20 wt%, which covers the full Ku band (12 to 18 GHz). The crystalline TiO_2_ composites show weaker microwave attenuation because of the less defects and oxygen-containing functional groups which induce the polarization losses. It is evidenced that the excellent impedance matching, the quarter-wavelength matching and superior dielectric losses coordinating around a same frequency are the main reasons for the excellent microwave absorption performance. This work would not only bring more inspiration that amorphous structures have better microwave absorption performance in designing high efficient microwave absorbers, but also provide a facile method in constructing and synthesizing high performance microwave absorption materials.

## Supplementary Materials

The following are available online at https://www.mdpi.com/2079-4991/10/11/2141/s1, Figure S1: FTIR spectra of a-T2 and c-T2. Figure S2: (a) Complex permittivity and (b) permeability of a-T2 with filler loading ratio of 10wt%, 15wt% and 20wt%. (c, d, e) Frequency-dependent reflection loss curve of a-T2 with filler loading ratio of 10wt%, 15wt% and 20wt%. Figure S3: 3D RL and 3D projection plots of (a, b) c-T1 and (c, d) c-T2. Figure S4: Complex permeability of a-T2 and c-T2: (a) real permeability, (b) imaginary permeability at 2-18 GHz. Figure S5: (a) Real permittivity and (b) imaginary permittivity of a-T1 and c-T12-18 GHz; (c) real permittivity and (d) imaginary permittivity of a-T3 and c-T3 at 2-18 GHz. Figure S6: Magnetic dissipation factor ($\mathrm{tg}\delta_{\mu }$) of a-T1, a-T2, a-T3 and c-T2. Figure S7: Frequency dependence of RL curves and the calculated matching thickness versus peak frequency of (a) a-T1, (b) a-T3, (c) c-T1 and (d) c-T2. Table S1: Different composites based on carbon materials for microwave absorption in recent researches.

## References

[1] Xu X.F., Shi S.H., Wan G.P., Hao C.C., He Z.Y., Wang G.Z. (2019). Uniformly Coating MnOx Nanoflakes onto Carbon Nanofibers as Lightweight and Wideband Microwave Absorbers with Frequency-selective Absorption. Mater. Des..

[2] Guo Y.F., Li J.Y., Meng F.B., Wei W., Yang Q., Li Y., Wang H.G., Peng F.X., Zhou Z.W. (2019). Hybridization-Induced Polarization of Graphene Sheets by Intercalation-polymerized Poly-aniline toward High Performance of Microwave Absorption. ACS Appl. Mater. Interfaces.

[3] Gao X., Luo C., Xue P., Zhang L., Wang Y., Zhang W. (2018). Synthesis of Hierarchical CuS/RGO/PANI/Fe_3_O_4_ Quaternary Composite and Enhanced Microwave Absorption Performance. J. Alloys Compd..

[4] Wu Y., Shu R.W., Zhang J.B., Sun R.R., Chen Y.N., Yuan J. (2019). Oxygen Vacancy Defects Enhanced Electromagnetic Wave Absorption Properties of 3D Net-like Multiwalled Carbon Nanotubes/Cerium Oxide Nanocomposites. J. Alloys Compd..

[5] Zhao Y., Wang J., Huang H., Cong T., Yang S., Pan L. (2020). Growth of Carbon Nanocoils by Porous α-Fe_2_O_3_/SnO_2_ Catalyst and Its Buckypaper for High Efficient Adsorption. Nano-Micro Letters.

[6] Yao Z., Zhou J., Lei Y., Lin H., Haidry A.A., Liu P. (2019). Synthesis and High-performance Microwave Absorption of Reduced Graphene Oxide/Co-doped ZnNi Ferrite/Polyaniline Composites. Mater. Lett..

[7] Shu R.W., Li W.J., Wu Y., Zhang J.B., Zhang G.Y. (2019). Nitrogen-doped Co-C/MWCNTs Nanocomposites Derived from Bi-metallic Metal-organic Frameworks for Electromagnetic Wave Absorption in the X-band. Chem. Eng. J..

[8] Xu X.F., Wang G.Z., Wan G.P., Shi S.H., Hao C.C., Tang Y.L., Wang G.L. (2020). Magnetic Ni/graphene Connected with Conductive Carbon Nano-onions or Nanotubes by Atomic Layer Deposition for Lightweight and Low-frequency Microwave Absorption. Chem. Eng. J..

[9] Singh S.K., Akhtar M.J., Kar K.K. (2018). Hierarchical Carbon Nanotube-Coated Carbon Fiber: Ultra Lightweight, Thin, and Highly Efficient Microwave Absorber. ACS Appl. Mater. Interfaces.

[10] Chen G.-X., Zhou Z., Li Q., Zhao Y. (2018). Coaxial Double-layer-coated Multiwalled Carbon Nanotubes toward Microwave Absorption. Mater. Lett..

[11] Wang H.G., Meng F.B., Huang F., Jing C.F., Li Y., Wei W., Zhou Z.W. (2019). Interface Modulating CNTs@PANi Hybrids by Controlled Unzipping of the Walls of CNTs to Achieve Tunable High-Performance Microwave Absorption. ACS Appl. Mater. Interfaces.

[12] Pan W., He M., Bu X., Zhou Y., Ding B., Huang T., Huang S., Li S. (2017). Microwave Absorption and Infrared Emissivity of Helical Polyacetylene@multiwalled Carbon Nanotubes Composites. J. Mater. Sci. Mater. Electron..

[13] Hou Z.L., Zhang K.L., Zhang J.Y., Bi S., Zhao Q.L. (2019). Multifunctional Broadband Microwave Absorption of Flexible Graphene Composites. Carbon.

[14] Yin X., Li M., Zhang L., Cheng L., Xu H., Zhu M., Wei H., Zhang H. (2019). Constructing Hollow Graphene Nanospheres Confined in Porous Amorphous Carbon Particles for Achieving full X Band Microwave Absorption. Carbon.

[15] Yu Q., Wang J., Xiong X.H., Yang S., Xu D.W., Chen P. (2019). 3D Nitrogen-doped Porous Magnetic Graphene Foam-supported Ni Nanocomposites with Superior Microwave Absorption Properties. J. Alloys Compd..

[16] Lin Y., Zong H., Yang H., Dong J., Wen B. (2018). Synthesis, Characterization, and Electromagnetic Wave Absorption Properties of Composites of Reduced Graphene Oxide with Porous LiFe_5_O_8_ Microspheres. ACS Sustain. Chem. Eng..

[17] Fu S., Xiao H., Huan G., Li N., Feng Q. (2019). Investigations on Structure-dependent Microwave Absorption Performance of Nano-Fe_3_O_4_ Coated Carbon-based Absorbers. Carbon.

[18] Zong M., Wang M., Zhang N., Huang Y., Liu X. (2019). Design and Microwave Absorption Properties of Thistle-like CoNi Enveloped in Dielectric Ag Decorated Graphene Composites. J. Colloid Interface Sci..

[19] Doley S., Agarwal V., Bora A., Borah D., Dolui S.K. (2019). Development of Sunflower Oil-Based Nonisocyanate Polyurethane/multiwalled Carbon Nanotube Composites with Improved Physico-chemical and Microwave Absorption Properties. Polym. Compos..

[20] MA R., FV H., AM M., HM M. (2019). Microwave Absorption of Polymer Nanocomposites on the Base High-density Polyethylene and Magnetite Nanoparticles. J. Elastomers Plast..

[21] Xie X., Sui G., Yang R., Jiang Y., Liu Y., Chen Y. (2018). Hierarchically Structured Cellulose Aerogels with Interconnected MXene Networks and their Enhanced Microwave Absorption Properties. J. Mater. Chem. C.

[22] Liu J., Zhang H.B., Xie X., Yang R., Liu Z., Liu Y., Yu Z.Z. (2018). Multifunctional, Superelastic, and Lightweight MXene/Polyimide Aerogels. Small.

[23] Fang X., Dong J., Sun Y., Zheng W., Jiang Z., Huang Y., Qian Y., Wei H. (2019). Ti_3_C_2_T_x_ MXene/polyaniline (PANI) Sandwich Intercalation Structure Composites Constructed for Microwave Absorption. Compos. Sci. Technol..

[24] Lv H., Guo Y., Yang Z., Cheng Y., Wang L.P., Zhang B., Zhao Y., Xu Z.J., Ji G. (2017). A Brief Introduction to the Fabrication and Synthesis of Graphene Based Composites for the Realization of Electromagnetic Absorbing Materials. J. Mater. Chem. C.

[25] Wang M., Huang Y., Zhang N. (2018). Synthesis of Graphene/thorns-like Polyaniline/alpha-Fe_2_O_3_@SiO_2_ Nanocomposites for Lightweight and Highly Efficient Electromagnetic Wave Absorber. J. Colloid Interface Sci..

[26] Wei J., Zang Z., Zhang Y., Wang M., Du J., Tang X. (2017). Enhanced Performance of Light-controlled Conductive Switching in Hybrid Cuprous Oxide/reduced Graphene Oxide (Cu_2_O/rGO) nanocomposites. Opt. Lett..

[27] Sun M., Wu F., Zeng Q., Xia Y., Xie A. (2018). The Effects of Annealing Temperature on the Permittivity and Electromagnetic Attenuation Performance of Reduced Graphene Oxide. Appl. Phys. Let..

[28] Wang Y., Fu Y., Wu X., Zhang W., Wang Q., Li J. (2017). Synthesis of Hierarchical Core-shell NiFe_2_O_4_@MnO_2_ Composite Microspheres Decorated Graphene Nanosheet for Enhanced Microwave Absorption Performance. Ceram. Int..

[29] Zhang X., Wang X., Sha P., Wang B., Ding Y., Du S. (2020). High-efficiency Electromagnetic Wave Absorption of Epoxy Composites Filled with Ultralow Content of Reduced Graphene/carbon Nanotube Oxides. Compos. Sci. Technol..

[30] Xu D., Yang S., Chen P., Yu Q., Xiong X., Wang J.J. (2019). Synthesis of Magnetic Graphene Aerogels for Microwave Absorption by an In-situ Pyrolysis. Carbon.

[31] Yu X., Wang L., Liu J., Xue S., Yang L., Li X., Zhang J., Xing L., Chen G., Wang M.J. (2019). Ferromagnetic Co_20_ Ni_80_ Nanoparticles Encapsulated Inside Reduced Graphene Oxide Layers with Superior Microwave Absorption Performance. J. Mater. Chem. C.

[32] Zhao H.B., Cheng J.B., Zhu J.Y., Wang Y.Z. (2019). Ultralight CoNi/rGO Aerogels toward Excellent Microwave Absorption at Ultrathin Thickness. J. Mater. Chem. C.

[33] Zhou X., Chuai D., Zhu D. (2019). Electrospun Synthesis of Reduced Graphene Oxide (RGO)/NiZn Ferrite Nanocomposites for Excellent Microwave Absorption Properties. J. Supercond. Novel Magn..

[34] Shu R., Zhang J., Wu Y., Wan Z., Zheng M. (2019). Facile Design of Nitrogen-doped Reduced Graphene Oxide/zinc Ferrite Hybrid Nanocomposites with Excellent Microwave Absorption in the X-band. Mater. Lett..

[35] Cui G., Lu Y., Zhou W., Lv X., Hu J., Zhang G., Gu G. (2019). Excellent Microwave Absorption Properties Derived from the Synthesis of Hollow Fe_3_O_4_@Reduced Graphite Oxide (RGO) Nanocomposites. Nanomaterials.

[36] Yin P., Zhang L., Li N., Wang J., Feng X., Wu W., Qi Y., Tao Y., Li H. (2019). Preparation of ZnO/Fe_3_O_4_/graphene Composite and Enhanced Microwave Absorption Performance in L-band. Mater. Technol..

[37] Xu H., Ai L., Yan J., Yan G., Zhang W. (2019). Enhanced Electrochemical Performance of LiNi_0. 5_Co_0. 2_Mn_0. 3_O_2_ Cathodes by Cerium Doping and Graphene Coating. Ceram. Int..

[38] Green M., Li Y., Peng Z., Chen X. (2020). Dielectric, magnetic, and microwave absorption properties of polyoxometalate-based materials. J. Magn. Magn. Mater..

[39] Tao F., Green M., Tran A.T.V., Zhang Y., Yin Y., Chen X. (2019). Plasmonic Cu_9_S_5_ nanonets for microwave absorption. ACS Appl. Nano Mater..

[40] Mo Z., Yang R., Lu D., Yang L., Hu Q., Li H., Zhu H., Tang Z., Gui X. (2019). Lightweight, Three-dimensional carbon Nanotube@TiO_2_ Sponge with Enhanced Microwave Absorption Performance. Carbon.

[41] Deng J., Li S., Zhou Y., Liang L., Zhao B., Zhang X., Zhang R. (2018). Enhancing the Microwave Absorption Properties of Amorphous CoO Nanosheet-coated Co (hexagonal and cubic phases) through interfacial polarizations. J. Colloid Interface Sci..

[42] Shen J., Yao Y., Liu Y., Leng J. (2019). Amorphous Bimetallic Nanowires with High-Performance Microwave Absorption: A Case for FeCo Nanowires. Nano.

[43] Kumar S., Verma N.K., Singla M.L. (2012). Size dependent reflective properties of TiO_2_ nanoparticles and reflectors made thereof. Dig. J. Nanomater. Bios..

[44] Ritter U., Scharff P., Siegmund C., Dmytrenko O., Kul-ish N., Prylutskyy Y.I., Belyi N., Gubanov V., Komarova L., Lizunova S. (2006). Radiation Damage to Multiwalled Carbon Nanotubes and Their Raman Vibrational Modes. Carbon.

[45] Zhang Y., Huang Y., Chen H., Huang Z., Yang Y., Xiao P., Zhou Y., Chen Y. (2016). Composition and Structure Control of Ultralight Graphene Foam for High-performance Microwave Absorption. Carbon.

[46] Choudhary V.R., Uphade B.S., Pataskar S.G. (2002). Low temperature complete combustion of dilute methane over Mn-doped ZrO_2_ catalysts: Factors influencing the reactivity of lattice oxygen and methane combustion activity of the catalyst. Appl. Catal. A Gen..

[47] Kuang B., Song W., Ning M., Li J., Zhao Z., Guo D., Cao M., Jin H. (2018). Chemical Reduction Dependent Dielectric Properties and Dielectric Loss Mechanism of Reduced Graphene Oxide. Carbon.

[48] Eigler S., Dotzer C., Hirsch A. (2012). Visualization of Defect Densities in Reduced Graphene Oxide. Carbon.

[49] Pei S., Cheng H.M. (2012). The Reduction of Graphene Oxide. Carbon.

[50] Shah A., Ding A., Wang Y., Zhang L., Wang D., Mu-Hammad J., Huang H., Duan Y., Dong X., Zhang Z. (2016). Enhanced Microwave Absorption by Arrayed Carbon Fibers and Gradient Dispersion of Fe Nanoparticles in Epoxy Resin Composites. Carbon.

[51] Cheng Y., Li Z., Li Y., Dai S., Ji G., Zhao H., Cao J., Du Y. (2018). Rationally Regulating Complex Dielectric Parameters of Mesoporous Carbon Hollow Spheres to Carry out Efficient Microwave Absorption. Carbon.

[52] Liu X., Nie X., Yu R., Feng H. (2018). Design of Dual-frequency Electromagnetic Wave Absorption by Interface Modulation Strategy. Chem. Eng. J..

[53] Qiao J., Zhang X., Xu D., Kong L., Lv L., Yang F., Wang F., Liu W., Liu J. (2020). Design and Synthesis of TiO_2_/Co/carbon Nanofibers with Tunable and Efficient Electromagnetic Absorption. Chem. Eng. J..

[54] Xuqiang J., Niu Y., Xu Y. (2019). Rational Design of Hierar-chical SiO_2_@TiO_2_ Composite with Large Internal Void Space for High-Performance Microwave Absorption. Russ. J. Phys. Chem. A.

[55] Xu J., Sun L., Qi X., Wang Z., Fu Q., Pan C. (2019). A Novel Strategy to Enhance the Multiple Interface Effect Using Amorphous Carbon Packaged Hydrogenated TiO_2_ for Stable and Effective Microwave Absorption. J. Mater. Chem. C.

[56] Singh S.K., Akhtar M., Kar K.K. (2019). Impact of Al_2_O_3_, TiO_2_, ZnO and BaTiO_3_ on the Microwave Absorption Properties of Exfoliated Graphite/epoxy Composites at X-band Frequencies. Compos. Part B.

[57] Yang Y., Xia L., Zhang T., Shi B., Huang L., Zhong B., Zhang X., Wang H., Zhang J., Wen G. (2018). Fe_3_O_4_@LAS/RGO Composites with a Multiple Transmission-absorption Mechanism and Enhanced Electromagnetic Wave Absorption Performance. Chem. Eng. J..

[58] Wu H., Qu S., Lin K., Qing Y., Wang L., Fan Y., Fu Q., Zhang F. (2018). Enhanced Low-Frequency Microwave Absorbing Property of SCFs@TiO_2_ Composite. Powder Technol..

[59] Wang Y., Lai Y., Wang S., Jiang W. (2017). Controlled Synthesis and Electromagnetic Wave Absorption Properties of Core-shell Fe_3_O_4_@SiO_2_ Nanospheres Decorated Graphene. Ceram. Int..

[60] Yang Z., Lv H., Wu R. (2016). Rational Construction of Graphene Oxide with MOF-derived Porous NiFe@C Nanocubes for High-performance Microwave Attenuation. Nano Res..

[61] Green M., Xiang P., Liu Z., Murowchick J., Tan X., Huang F., Chen X. (2019). Microwave absorption of aluminum/hydrogen treated titanium dioxide nanoparticles. J. Mater..

[62] Green M., Van Tran A.T., Smedley R., Roach A., Murowchick J., Chen X. (2019). Microwave absorption of magnesium/hydrogen-treated titanium dioxide nanoparticles. Nano Mater. Sci..

[63] Wen B., Cao M., Lu M., Cao W., Shi H., Liu J., Wang X., Jin H., Fang X., Wang W. (2014). Reduced Graphene Oxides: Light-weight and High-efficiency Electromagnetic Interference Shielding at Elevated Temperatures. Adv. Mater..

[64] Mathew G., Dey P., Das R., Chowdhury S.D., Paul Das M., Veluswamy P., Neppolian B., Das J. (2018). Direct Electrochemical Reduction of Hematite Decorated Graphene Oxide (alpha-Fe_2_O_3_@rGO) Nanocomposite for Selective Detection of Parkinson’s Disease Biomarker. Biosens. Bioelectron..

[65] Molina J., Cases F., Moretto L.M. (2016). Graphene-based Materials for the Electrochemical Determination of Hazardous Ions. Anal. Chim. Acta.

[66] Huang Y., Li H., Wang Z., Zhu M., Pei Z., Xue Q., Huang Y., Zhi C. (2016). Nanostructured Polypyrrole as a Flexible Electrode Material of Supercapacitor. Nano Energy.

[67] Xia T., Zhang C., Oyler N.A., Chen X. (2013). Hydrogenated TiO_2_ Nanocrystals: A Novel Microwave Absorbing Material. Adv. Mater..

[68] Cole K.S., Cole R.H. (1941). Dispersion and absorption in Dielectrics I. Alternating current characteristics. J. Chem. Phys..

[69] Sun X., He J., Li G., Tang J., Wang T., Guo Y., Xue H. (2013). Laminated Magnetic Graphene with Enhanced Electromagnetic Wave Absorption Properties. J. Mater. Chem. C.

[70] Zhao Y., Zhang H., Yang X., Huang H., Zhao G., Cong T., Zuo X., Pan L. (2020). In situ construction of hierarchical core–shell Fe_3_O_4_@C nanoparticles–helical carbon nanocoil hybrid composites for highly efficient electromagnetic wave absorption. Carbon.

[71] Song C., Yin X., Han M., Li X., Hou Z., Zhang L., Cheng L. (2017). Three-dimensional Reduced Graphene Oxide Foam Modified with ZnO Nanowires for Enhanced Microwave Absorption Properties. Carbon.

[72] Wu T., Liu Y., Zeng X., Cui T., Zhao Y., Li Y., Tong G. (2016). Facile Hydrothermal Synthesis of Fe_3_O_4_/C Core–shell Nanorings for Efficient Low-frequency Microwave Absorption. ACS Appl. Mater. Interfaces.

[73] Yin Y., Liu X., Wei X., Yu R., Shui J. (2016). Porous CNTs/Co Composite Derived from Zeolitic Imidazolate Framework: A Light-weight, Ultrathin, and Highly Efficient Electromagnetic Wave Absorber. ACS Appl. Mater. Interfaces.

[74] Tong G., Liu F., Wu W., Du F., Guan J. (2014). Rambutan-like Ni/MWCNT Heterostructures: Easy Synthesis, Formation Mechanism, and Controlled Static Magnetic and Microwave Electromagnetic Characteristics. J. Mater. Chem. A.

[75] Quan B., Liang X., Ji G., Cheng Y., Liu W., Ma J., Zhang Y., Li D., Xu G. (2017). Dielectric Polarization in Electromagnetic Wave Absorption: Review and Perspective. J. Alloys Compd..

[76] Qiu X., Wang L., Zhu H., Guan Y., Zhang Q. (2017). Light-weight and Efficient Microwave Absorbing Materials Based on Walnut Shell-derived Nano-porous Carbon. Nanoscale.

[77] Xu H., Yin X., Zhu M., Han M., Hou Z., Li X., Zhang L., Cheng L. (2017). Carbon Hollow Microspheres with a Designable Mesoporous Shell for High-performance Electromagnetic Wave Absorption. Appl. Mater. Interfaces.

[78] Wang X.X., Ma T., Shu J.C., Cao M.S. (2018). Confinedly Tailoring Fe_3_O_4_ Clusters-NG to Tune Electromagnetic Parameters and Microwave Absorption with Broadened Bandwidth. Chem. Eng. J..

[79] Xia X., Zhong Z., Weng G. (2017). Maxwell–Wagner–Sillars Mechanism in the Frequency Dependence of Electrical Conductivity and Dielectric Permittivity of Graphene-polymer Nanocomposites. Mech. Mater..

[80] Song Q., Ye F., Yin X., Li W., Li H., Liu Y., Li K., Xie K., Li X., Fu Q. (2017). Carbon Nanotube-multilayered Graphene Edge Plane Core-shell Hybrid Foams for Ultrahigh-performance Electromagnetic-Interference Shielding. Adv. Mater..

[81] Yao D., Li T., Zheng Y., Zhang Z. (2019). Fabrication of a Functional Microgel-based Hybrid Nanofluid and Its Application in CO_2_ Gas Adsorption. React. Funct. Polym..

