# Supplementary material for: A Facile Synthesis of Novel Amorphous TiO2 Nanorods Decorated rGO Hybrid Composites with Wide Band Microwave Absorption
Source: Nanomaterials (Basel). 2020 Oct 27;10(11):2141. doi: 10.3390/nano10112141 (PMC7692450; doi:10.3390/nano10112141)
Supplement: Supplementary file 1 [file nanomaterials-10-02141-s001.pdf]

nanomaterials

Supporting Information

Facile Synthesis of Novel Amorphous TiO_2 Nanorods Decorated rGO Hybrid Composites with Wide Band Microwave Absorption

Hao Zhang [1], Yongpeng Zhao [12], Xuan Yang [3], Guolin Zhao [4], Dongmei Zhang [1], Hui Huang [1], Shuaitao Yang [1], Ningxuan Wen [1], Zeng Fan [1] and Lujun Pan [1*].

[1] School of Physics, Dalian University of Technology, Dalian, Liaoning 116024, China.
[2] School of Microelectronics, Dalian University of Technology, Dalian, Liaoning 116024, China.
[3] School of Materials Science and Engineering, Dalian University of Technology, Dalian, Liaoning 116024, China.
[4] School of Physics and Astronomy, Shanghai Jiao Tong University, Shanghai 200240, China.

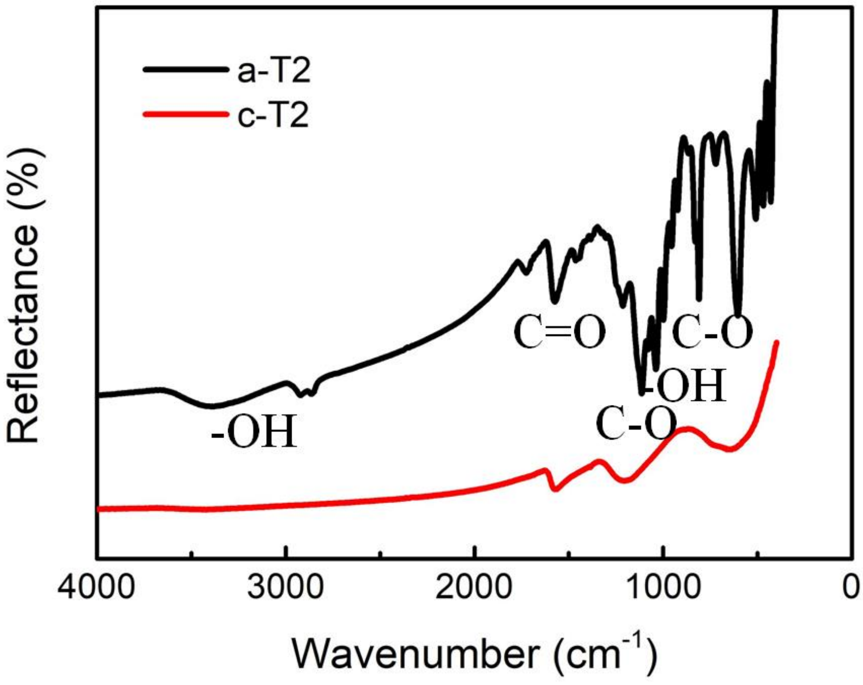

Figure S1. FTIR spectra of a-T2 and c-T2.

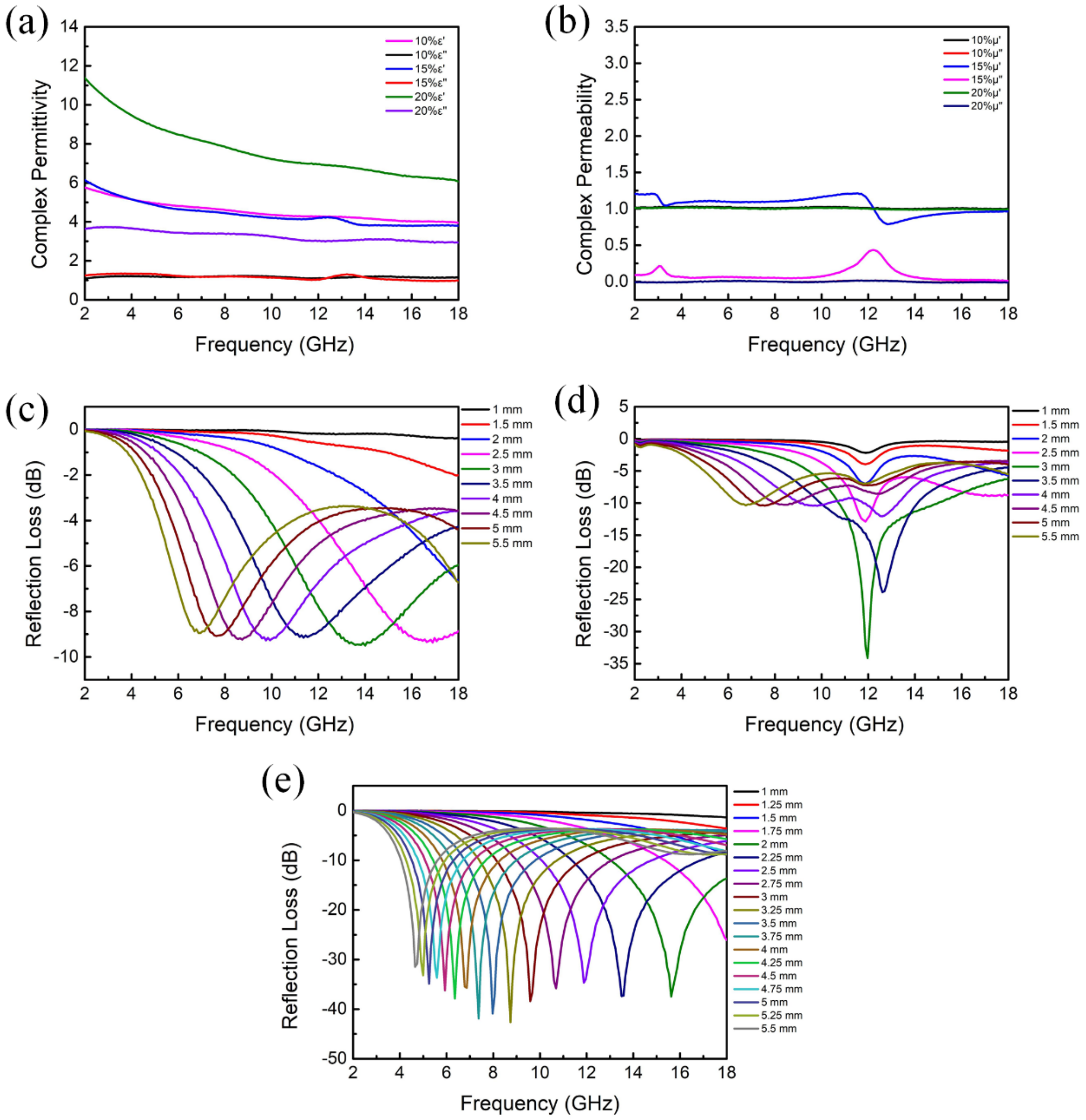

Figure S2. (a) Complex permittivity and (b) permeability of a-T2 with filler loading ratio of 10wt%, 15wt% and 20wt%. (c, d, e) Frequency-dependent reflection loss curve of a-T2 with filler loading ratio of 10wt%, 15wt% and 20wt%.

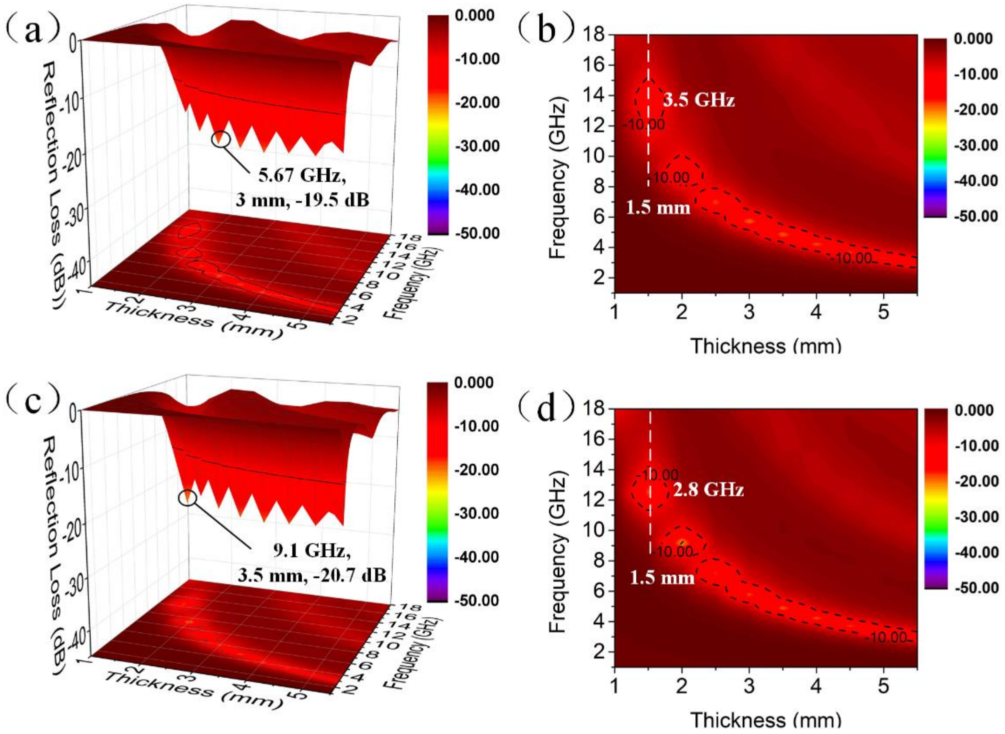

Figure S3. 3D RL and 3D projection plots of (a, b) c-T1 and (c, d) c-T2.

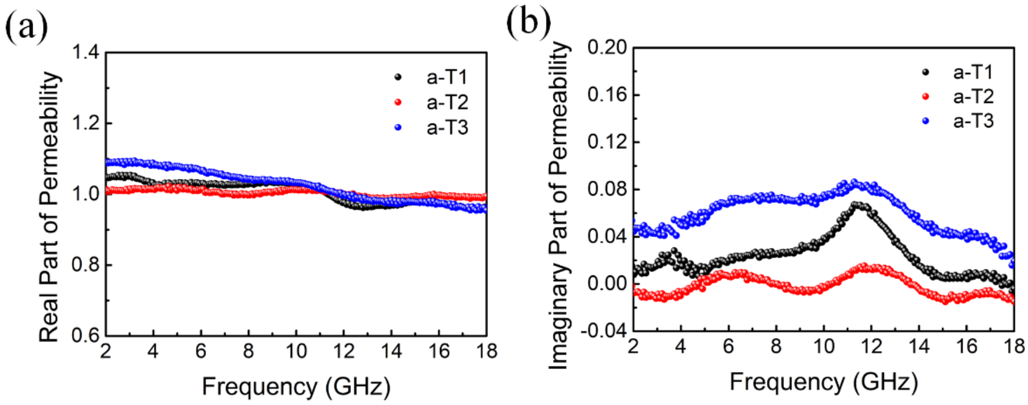

Figure S4. Complex permeability of a-T2 and c-T2: (a) real permeability, (b) imaginary permeability at 2-18 GHz.

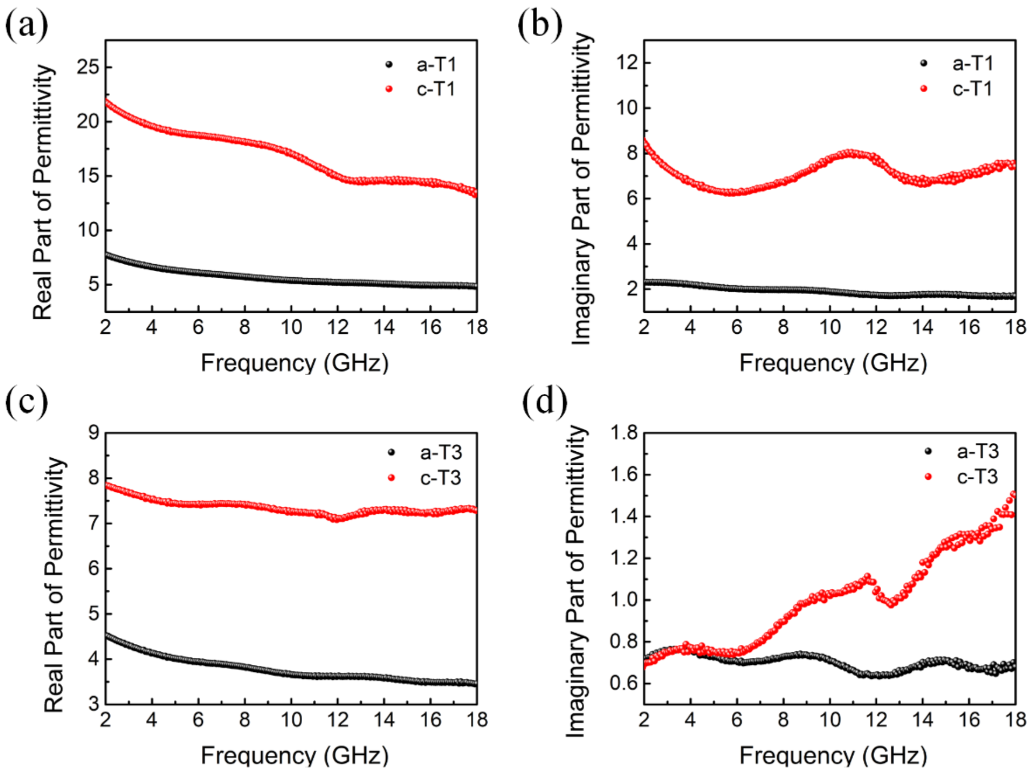

Figure S5. (a) Real permittivity and (b) imaginary permittivity of a-T1 and c-T12-18 GHz; (c) real permittivity and (d) imaginary permittivity of a-T3 and c-T3 at 2-18 GHz.

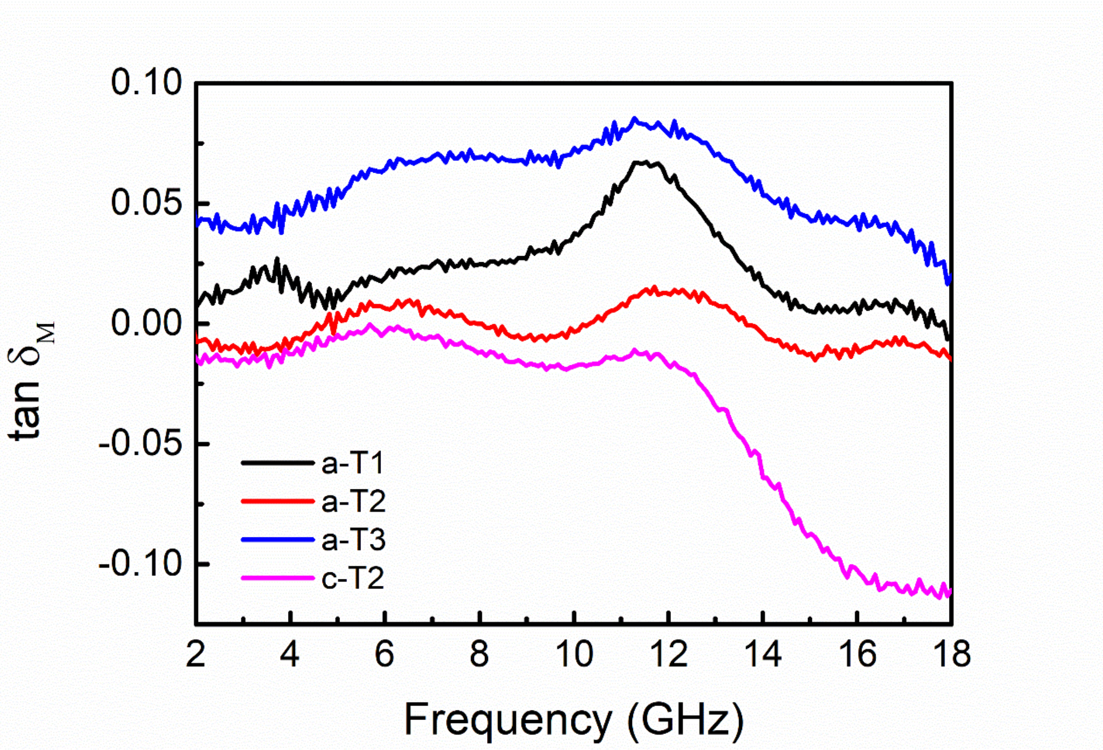

Figure S6. Magnetic dissipation factor (tgδ_μ) of a-T1, a-T2, a-T3 and c-T2.

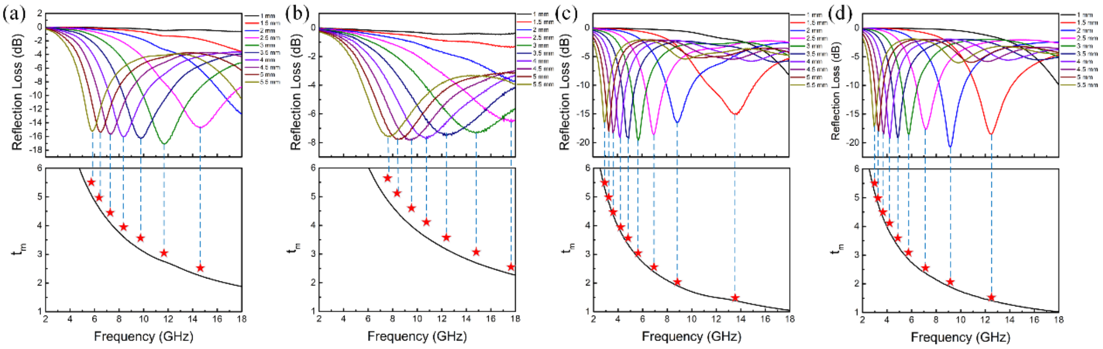

Figure S7. Frequency dependence of RL curves and the calculated matching thickness versus peak frequency of (a) a-T1, (b) a-T3, (c) c-T1 and (d) c-T2.

Table S1. Different composites based on carbon materials for microwave absorption in recent researches.

Absorbers	Matrix	Loading ratio (wt%)	Thickness (mm)	RL_{min} (dB)	Effective absorption bandwidth (GHz)	refs
**CNT/TiO_2 sponge**	Paraffin	30	2	-31.8	2.76	[46]
**TiO_2/Co/CNFs**	Paraffin	20	3.5	-50	5.2	[59]
**Fe_3O_4@SiO_2/rGO nanocomposites**	Paraffin	50	1.5	-26.4	2.6	[65]
**B-TiO_2/C**	Paraffin	50	1.5	-73.2	6.8	[61]
**TiO_2/EG**	Epoxy	30	1.4	-33.9	2.7	[62]
**SiO_2@TiO_2 nanoparticle**	Paraffin	50	3	-35.2	2.5	[60]
**SCFs@TiO_2**	Paraffin	30	1	-46.3	2.4	[64]
**NiFe@C@GO**	Silicon resin	30	2.8	-51	4.55	[66]
**Fe_3O_4@LAS/rGO**	Paraffin	50	2.1	-65	4	[63]
**Amorphous TiO_2/rGO composites**	Paraffin	20	3.25	-42.8	6.2	This work
